# *Caenorhabditis elegans* as a Prediction Platform for Nanotechnology-Based Strategies: Insights on Analytical Challenges

**DOI:** 10.3390/toxics11030239

**Published:** 2023-03-01

**Authors:** Priscila Gubert, Greici Gubert, Ronei Cardoso de Oliveira, Isabel Cristina Oliveira Fernandes, Iverson Conrado Bezerra, Bruna de Ramos, Milena Ferreira de Lima, Daniela Teixeira Rodrigues, Adriana Farias Nunes da Cruz, Ernesto Chaves Pereira, Daiana Silva Ávila, Dante Homero Mosca

**Affiliations:** 1 Keizo Asami Institute, iLIKA, Federal University of Pernambuco, Recife 50670-901, Brazil; 2 Graduate Program in Biology Applied to Health, PPGBAS, Federal University of Pernambuco, Recife 50670-901, Brazil; 3 Graduate Program in Pure and Applied Chemistry, POSQUIPA, Federal University of Western of Bahia, Bahia 47808-021, Brazil; 4 Postdoctoral Program in Chemistry, Federal University of São Carlos, São Carlos 13565-905, Brazil; 5 Postdoctoral Program in Physics, Federal University of São Carlos, São Carlos 13565-905, Brazil; 6 Oceanography Department, Federal University of Pernambuco, Recife 50670-901, Brazil; 7 Graduate Program in Biological Sciences, Toxicological Biochemistry, Federal University of Santa Maria, Santa Maria 97105-900, Brazil; 8 Graduate Program in Biochemistry, Federal University of Pampa (UNIPAMPA), Uruguaiana 97501-970, Brazil; 9 Postdoctoral Program in Physics, Federal University of Paraná, Curitiba 80060-000, Brazil

**Keywords:** toxicology, nanomaterials, nanosystems, nanotoxicity, biomedical applications, environmental toxicology

## Abstract

Nanotechnology-based strategies have played a pivotal role in innovative products in different technological fields, including medicine, agriculture, and engineering. The redesign of the nanometric scale has improved drug targeting and delivery, diagnosis, water treatment, and analytical methods. Although efficiency brings benefits, toxicity in organisms and the environment is a concern, particularly in light of global climate change and plastic disposal in the environment. Therefore, to measure such effects, alternative models enable the assessment of impacts on both functional properties and toxicity. *Caenorhabditis elegans* is a nematode model that poses valuable advantages such as transparency, sensibility in responding to exogenous compounds, fast response to perturbations besides the possibility to replicate human disease through transgenics. Herein, we discuss the applications of *C. elegans* to nanomaterial safety and efficacy evaluations from one health perspective. We also highlight the directions for developing appropriate techniques to safely adopt magnetic and organic nanoparticles, and carbon nanosystems. A description was given of the specifics of targeting and treatment, especially for health purposes. Finally, we discuss *C. elegans* potential for studying the impacts caused by nanopesticides and nanoplastics as emerging contaminants, pointing out gaps in environmental studies related to toxicity, analytical methods, and future directions.

## 1. Introduction

Nanotechnology-based strategies enhance the possibilities over matters already used, allowing the development of new properties. The improvements benefit many fields, including the food, health, environment, agriculture, and electronics industries. Nanotechnology has multifaceted applications in the health field. For instance, improving the efficiency of drugs, decreasing their toxicity, enabling slow and prolonged drug delivery, and enhancing diagnosis and vaccine production.

In particular, nanoparticles can exchange specific design features (e.g., targeting antibodies, the encapsulated drug, and control over how/when the diseased site interacts with this drug) in a plug-and-play format to treat diseases. Beyond their potential as delivery system platforms, pharmaceuticals, nanomedicines, and healthcare therapies highly depend on emerging formulation technologies in the foreseeable future [1,2,3].

Otherwise, many products under environmental context turn into fragmented pieces liable to be uptaken by animals, microorganisms, plants, and in the worst perspective, already happening, by humans. This could trigger and/or enhance the risks of toxic impairments.

In order to determine the properties and drive the targeting of each new nanomaterial, they need to be scientifically tested to guarantee both safety and effectiveness. Many experimental models have been proposed, including the alternative invertebrate *Caenorhabditis elegans*. *C. elegans* is a non-pathogenic worm that allows the observation of nanomaterial effects in a few days due to its short life cycle and lifespan. The small size (~1 mm as an adult) is perfect for performing experiments with a small amount of nanomaterial samples. Moreover, its transparency provides the possibility of the image acquisition, in vivo, of dyed nanomaterials. The transgenics can be used to test human conditions as well as predict environmental impacts.

Here, we describe important results on nanotechnology-based strategies tested in *C. elegans*. We also bring tips from our team’s experiments, actively working with the nematode and nanopesticides, nanoplastics, carbon nanosystems, and magnetic nanoparticles (MNPs). In addition, we discuss the future perspectives in this field, such as regulation and the insertion of this animal model in environmental risk assessments.

## 2. Nanomaterials

Nanoscale materials or “nanomaterials” are unique nano-objects, generally defined as having at least one dimension between 1 and 100 nanometers, presenting unique optical, electronic, magnetic, mechanical, thermophysical, or biochemical properties.

Nanoparticles and nanostructured materials, as well as their assemblies in nanosystems, are incidentally found in nature or deliberately designed and engineered by human manufacturing. They exhibit distinct performances compared to their mass counterparts, behaving as building blocks for innovations and disruptive breakthroughs over the last three decades [4,5].

### 2.1. An Overview

Historically, over millennia, nanoparticles have been produced from volcanic eruptions, forest fires, dust storms, simple erosion, and human activities. Besides, nanoparticles and nanostructures are also present in living organisms (microorganisms, bacteria, algae, viruses, and complex organisms, including humans). In the industrial era, the proliferation of nanoparticles derived from engine exhaust, mining activities, demolition of buildings, additives in lubricants, and cosmetic products has profoundly increased anthropogenic particulate dissemination, increasing the potential risks for living organisms.

In research and industry, inorganic nanoparticles are engineered and manufactured from a metal, metal oxide, and semiconductor materials, as well as ceramic composites. Nanomaterials can be manufactured by different routes, including physical (e.g., gas-phase deposition, power ball milling, pulsed laser ablation, and laser ablation synthesis in solutions), chemical (e.g., combustion, chemical co-precipitation, thermal decomposition, microemulsion, and hydrothermal synthesis), and biological (e.g., bacteria, plant extracts, and protein-mediated) methods. The choice of the method determines the quantity of synthesized nanoparticles and the control of the desired morphological and microstructural properties (size, shape, structure, colloidal stability, and physicochemical properties) [6,7,8]. Both inorganic and organic nanoparticles have been proposed for different applications.

However, synthesizing nanoparticles in a suitable amount for biomedical testing is still challenging. Synthetic and natural organic nanoparticles have increased interest due to their potential applications in many fields of technology and in biological applications, such as active pharmaceutical agents, nutrients, cosmetics, dyes, and inks, among others [4,5].

Recent advances in molecular modeling, automated chemical synthesis, and specific functionalization processes envisaging biological tagging and targeting have contributed to the development of drug delivery, therapies, and diagnostics. Both top-down and bottom-up approaches have been used to obtain drug nanoformulations and develop new nanoagents for tumor treatments, diagnostics, and imaging [5].

Despite several strategies in this roadmap, challenges associated with post-formulation (e.g., stability, solubility, clearance, cytotoxicity, and genotoxicity) make successful use in industrial applications difficult once following the regulatory issues. Notably, many potential pharmaceutical drugs fail to proceed to clinical trials due to their poor solubility in water [9,10]. However, there are different propositions to overcome this issue once nanosizing by itself leads to a significant increase in water solubility [11].

Recent developments based on lipid and other biologically complex nanoparticles show promising results in suitable levels for specific and personalized drug developments [12]. Besides, the increasing number of nanodrugs and nanoagents for therapeutic, and diagnostic purposes (including fluorescent or radioactive nanoparticles and traceable contrast enhancers) approved by regulatory agencies in the last two decades is responsible for significant advances in medicine.

In particular, theranostics consolidates as an innovative concept that integrates therapy and diagnosis into a single platform using nanomaterials in modern medicine [13,14]. Theranostics enables efficient imaging diagnostics and pharmacotherapy, providing a transition from conventional medicine to personalized medicine with specific and economical treatment protocols.

Some important classes of nanoparticles responsible for applications in medicine are shown in Table 1.

Despite the well-recognized advantages of nanotechnology-based strategies, only a few examples successfully achieved the market, including Doxil^®^, Caelyx^®^, Myocet^®^, and Abraxane^®^ [16].

An increasing number of studies report nanoparticles being used to target bacteria as an alternative to antibiotics. However, the antibacterial mechanisms of nanoparticles still need to be understood entirely. The mechanisms currently accepted include reactive oxidative stress induction, metal ion release, and non-oxidative ones. Besides, multiple mechanisms of simultaneous action can also occur, making it difficult for bacterial cells to resist nanoparticles [17]. In the case of metallic nanoparticles, size, surface charge, and morphology concomitant with a crystal structure are crucial factors affecting the antibacterial action. Still, environmental conditions and exposure time are other significant factors [17]. Considering the material transport and diffusion, endocytosis and simple traffic of nanoparticles with diameters in the range of 1–10 nm through the bacterial cell membrane are the main mechanisms [18,19].

As significant ingredients for the future developments of drugs in medicine, nanoparticles also promise advances in health therapies and diagnostics. The use of nanoparticles in growing fields such as pharmacogenetics, proteomics, and biomarker profiling forms the backbone of new medical care and treatments. For this reason, it is crucial to master the production and functionalization of nanoparticles to carry out the transport of labeled drugs, the targeted delivery of drugs, the generation of images from traceable biomarkers, and the application of theranostic treatments. Nanoformulations based on synthetic metal and oxide nanoparticles, as well as polymeric nanoparticles, quantum dots, and carbon nanostructures, are now administered alone or in combination with naturally occurring organic nanoparticles (e.g., liposomes, exosomes, and dendrimers), which add other applications as they are responsible for a wide variety of biological functionalities in biological systems [20,21,22].

### 2.2. Nanotoxicity

The benefits of nanoparticles for medicine are numerous, but the dissemination of nanoparticles in living ecosystems brings severe challenges due to nanotoxicity, cytotoxicity, and genotoxicity.

A direct permeation of nanoparticles through the plasmatic membrane occurs concomitantly with transient property modifications in the cell’s selectively permeable regulation and transmembrane protein channels and transporters. Like viruses, nanoparticles can enter cells and interact with subcellular structures. Several nano-sized materials can cross cellular membranes by passive pathways such as diffusion and adhesive interactions. Thermal capillary and surface tension also play a significant role in controlling the entry of nanoparticles into cells. These properties described before, size, shape, solubility, and surface chemistry, are responsible for the ability of these compounds to enter the cell. But despite long-standing studies [23], it is still to be elucidated how these particles’ properties act on cell membranes. As the flat surface of material changes to nanoparticles (eventually approaching the size of proteins themselves), cellular responses to materials in a biological environment change dramatically. The protein-particle interaction is often represented as a protein crown on a nanoparticle [24].

Animal and human studies show that inhaled nanoparticles are less efficiently removed than larger particles in the lungs by the macrophage clearance mechanisms, causing lung damage. Regarding nanoparticle size dependence in respiratory system exposures, the predicted fractional deposition of inhaled nanoparticles in the nasopharyngeal, tracheobronchial, and alveolar regions of the human respiratory tract during nose breathing, reveals that, for 1 nm particles, 90% are deposited in the nasopharyngeal compartment, approximately 10% in the tracheobronchial region, and almost none in the alveolar region. Comparatively, 5 nm particles show about the equal deposition of roughly 30% of the inhaled particles in all three regions, whereas 20 nm particles have the highest deposition efficiency in the alveolar region (~50%) with deposits efficiencies of approximately 15% in tracheobronchial and nasopharyngeal regions [25].

The absorption of metal oxide nanoparticles in the gastrointestinal tract depends on the particle’s size, with the uptake diminishing for larger particles [26]. Metal nanoparticles coated with phytochemicals often have low nanotoxicity and serve bioapplications such as antibacterial, antimicrobial, and antioxidant activities, as well as a contrast agent for X-ray imaging [27]. In contrast, several metal oxide nanoparticles exhibit dose-dependent cytotoxic and mutagenic effects, which are especially sensitive to oxidative stress [28,29]. Metal and metal oxide nanoparticles, including titanium dioxide nanoparticles (TiO_2_–NPs), zinc oxide nanoparticles (ZnO–NPs), and silicon dioxide nanoparticles (SiO_2_–NPs), have a wide range of commercial and ecotoxicological applications and effects. These NPs are used in industrial production and consumer products; however, their persistent chemical composition and lack of biodegradability make their presence in the environment a cause for concern. Studies have been conducted to assess the toxicity of these NPs at environmentally relevant concentrations, with detrimental effects detected in ZnO–NPs and TiO_2_–NPs at 0.05 μg L^−1^ and SiO_2_–NPs at 5 μg L^−1^. The order of toxicity was determined to be ZnO–NPs > TiO_2_–NPs > SiO_2_–NPs. Furthermore, reactive oxygen species’ production correlated with lethality, growth, reproduction, and locomotion [30]. Additionally, exposure to 10 μM of metals, except Cu, Zn, and Ag, reduced associative learning at 5 and 18 h. 2.5 and 10 μM of metals did not affect body bend or thermotaxis. Pb and Hg were more toxic than other metals, causing severe toxicity on associative learning, thermotaxis, and locomotion [31].

Efficient nanoparticle uptake and scavenging have been intensively studied for decades, elucidating mechanisms of particle targeting and nanoparticle therapeutics. In particular, macrophages are among the first and primary cell types that process nanoparticles and promote their internalization [32]. Still, nanoparticles can also be translocated through the circulatory, lymphatic, and nervous systems to many tissues and organs, including the brain [33].

Many nanoparticles are widely distributed in the air or soil and can be ingested by bacteria and enter our food chain, resulting in higher concentrations in organisms higher up the food chain [25,34]. The uptake, translocation, and accumulation of nanomaterials in plant cells or parts and their recognized ways of phytotoxicity are not less significant [35]. Indeed, many papers have demonstrated that manufactured nanoparticle phytotoxicity can cause severe biochemical and genetic impacts through the production of ROS. This occurs via oxidative stress, lipid peroxidation, and protein and DNA damage in plants. From a different point of view, while many nanomaterials cause toxic effects, many act as growth regulators, inducing significant increases in biomass and even nutritional quality. Therefore, nanoparticles are promising for use as fertilizers in agriculture. However, there is a strong need for studies of the influence of nanoparticles on life cycles at different trophic levels in ecosystems.

Despite efforts of several government organizations worldwide, there is no specific international regulation, no international agreement upon protocols or legal definitions for production, handling or labeling, testing toxicity, and evaluating the environmental impact of nanoparticles from a safety perspective [4,36].

### 2.3. Assessment and Regulation

Regulatory scientific research and monitoring of scientific and technological advances are crucial for the beneficial convergence of nanotechnological products and healthcare developments. Translation of a typical nanomedicine platform application from the discovery stage to approval and commercialization must pass a long track of pre-clinical studies and human clinical trials. Only the strategy of strengthening regulatory science research in nanotechnology ensures the safety, efficacy, and quality of drugs, vaccines, as well as other biological products and medical devices for human and animal health.

The coronavirus disease (COVID-19) vaccines have recently been developed within new vaccine platforms using synthetic lipid nanoparticle components to encapsulate and protect messenger RNA [4]. Even though the development of universal vaccines capable of generating a long-lasting protective immune response without using live pathogens remains a challenge, the so-called “nanoparticle in clinic” is entering a new phase due to its tremendous global impact.

According to the Food and Drugs Agency (FDA) in the U.S. and the European Medicines Agency (EMA), until 2020, more than 30 nanoparticles from clinical trials were approved (including 20 lipid-based, 10 inorganic, and 2 protein-based nanoparticles), whereas 100 yet-unapproved nanoparticles are being evaluated in clinical trials [3]. Therefore, there has been a massive increase in the clinical introduction of new nanoparticle technologies, and many are promising candidates for further advances and gains regarding biocompatibility and targeting ability. A snapshot of the different parameters and strategies that are iteratively optimized for desired performances of clinically approved nanoparticles reveals: (a) nanoparticle sizes from 40 nm to 250 nm and drug load weight < 30% with an initial release rate < 16% per hour [3], (b) amphiphilic polyethylene glycol (PEG) coatings on nanoparticles to shield the surface from aggregation and to improve systemic circulation time and decrease immunogenicity [37], (c) molecule inhibitor targeting (ACUPA) per nanoparticle < 1000 [3], (d) polymeric polylactic acid (PLA)—based coating for biocompatible hydrolytic degradation [38], and (d) encapsulation processes preferably by emulsion and nanoprecipitation [1,2,3].

Although current drug delivery systems through nanoparticles have been successfully used in the clinic, intrinsically foreign nanomaterials have potential toxicity. More studies are required to increase biocompatibility with multifunctionality, and the parent cells of natural nano-sized structures combined with synthetic nanoparticles to achieve site-specific drug delivery and improve diagnostics and therapies.

Over these past decades, newer generations of nanoparticles (about) have emerged capable of performing additional delivery functions that enable treatment via new therapeutic modalities.

In this sense, model nanotoxicity studies based on non-mammalian alternatives to the *C. elegans* platform are rather promising for technological advances and regulatory scientific research for handling, labeling, testing toxicity, and evaluating the environmental impact of nanoparticles [39].

## 3. *Caenorhabditis elegans*

The free-living nematode *Caenorhabditis elegans* is a simple invertebrate organism that has gained scientific and industrial attention due to the wide possibilities that the model provides. *C. elegans* has been a relevant model for neurobiology and developmental biology since the 1960s, and so far, cell fate, neuronal development, and the connectome have been unraveled. The worm has 959 somatic cells; among them, 302 are neurons (in hermaphrodites). The anatomy of *C. elegans* includes the following systems: an alimentary system, a reproductive system, a nervous system, and an excretory system, all protected by the cuticle [40]. 

Particularly in nanotoxicology, some features are important for its successful application in this field [41]. It is a small and non-parasitic worm, therefore it has a low cost maintenance, making possible to grow billions of worms in a small space and the amount of nanoparticles needed to be tested are much lower in comparison to other in vivo models; the life and reproductive cycles are short, which allows for the observation of lifespan alterations, reproductive damage and transgenerational effects in less than one month [41,42,43]; their genome is fully sequenced and it is known that *C. elegans* conserves a genetic identity with human genes of 41.7% (20–71% in a range) and several cellular processes are highly similar between the species, therefore making possible to extrapolate the findings from an invertebrate to mammals [44]; the easy genetic manipulation that allows the construction of deletion mutants or fluorescent tagged proteins to investigate the toxicity mechanism of the nanoparticles [45]; they are transparent, which facilitates the visualization of the organs, nanoparticles internalization and cellular markers [46]. Notably, this nematode is extremely sensitive to the adverse effects of the environment where it is found, changing its morphology, behavior, and cellular redox state [47]. This makes it possible to use the nematode as a bioindicator of the environmental impact of nanomaterials.

Recently, the fast development of nanoparticles has not been accompanied by their safety assessment. It is urgent that their impact on the environment and human health be investigated and understood. *C. elegans* can be used in ecotoxicological evaluations due to its presence in several ecological systems [48] and also in toxicology due to the similarity of several systems with humans [49,50]. In this sense, an increasing number of studies have applied *C. elegans* as a model, and a relevant amount of evidence supports its insertion in environmental risk assessments [51].

Besides, new methodologies to study nanoscience have been developed in *C. elegans*. For instance, given the difficulty of determining the nanoparticles fate, methods such as transmission electron microscopy (TEM, hyperspectral dark field microscopy (HDFM), or synchrotron radiation X-ray fluorescence (μ-SRXRF) can help to identify, locate, and characterize individual nanoparticles in specific regions of the body of small animals. Combined with mass spectroscopy, it is also possible to investigate the in vivo degradation of nanoparticles [52]. We will next address the several applications of *C. elegans* in nanotoxicology, including the newest data obtained from recent studies and the techniques used to study nanoparticle safety in this animal model.

## 4. *Caenorhabditis elegans* and Magnetic Nanoparticles

MNPs are nanometric materials that can respond to and be manipulated by an external magnetic field. Such properties allow them to be used in separation techniques such as cell separation and water purification [7,12]. Their magnetic and surface properties grant this class of materials a high interest in drug and gene delivery, cell labeling, traceable biological labeling, and magnetic hyperthermia as adjunctive therapy. Drugs can be carried on the surface of the MNPs by conjugation, or a magnetic hollow nanoparticle can be used to incorporate drugs and biomolecules [53,54]. Due to the low toxicity and high biocompatibility of some MNPs, they are very promising to be used as a platform for drug delivery in biological systems [54].

Usually, the magnetic nanoparticles used as drug carriers are composed of a magnetic core coated with a suitable biocompatible layer. This coating provides a surface for the conjugation of drug molecules and targeting ligands, protects the core from oxidation, increases biocompatibility, and reduces the tendency of the nanoparticles to aggregate, improving their dispersibility and colloidal stability [54,55]. Furthermore, the magnetic core allows the accumulation of nanoparticles in targeted areas using an external magnetic field. These properties make MNPs highly attractive for in vivo applications such as MRI [56,57].

Different types of magnetic nanoparticles have allowed MRI to be a widely used and powerful tool for non-invasive clinical diagnosis due to its high degree of soft tissue contrast, spatial resolution, and penetration depth. Similarly, these nanoparticles can be used for magnetic hyperthermia to selectively ablate abnormal cells by exposing biological tissues to high temperatures, allowing for the thermal shock intensity to lead the cells to undergo necrosis or activate programmed cell death (apoptosis). This method of magnetic hyperthermia has the benefit of bypassing the limitation of penetration depth posed by light irradiation hyperthermia methods, while its disadvantage is that it directs the alternating magnetic field toward the entire body instead of focusing on the tumor itself. Some other challenges are the inhomogeneous distribution of nanoparticles through the tissue for uniform heating, the inability to accurately perform thermal dosimetry, and the dependence of heating efficiency on thermal dose rate.

Ideally, for hyperthermia treatment, a small quantity of MNPs should be able to generate enough heat when exposed to an external magnetic field. For this reason, much research has been dedicated to finding MNPs with excellent heating capability.

The efficiency of conversion of absorbed alternating magnetic field energy to heat by a magnetic nanoparticle is described by its specific absorption rate (SAR). Heat generation can drastically decrease when the nanoparticles are placed in a cellular environment. It has been reported that a systematic fall, at least half the initial value in solution, in the heating capacity of nanoparticles occurs as soon as they attach to the cell membrane or are internalized [58]. In biological systems, the intracellular degradation and, mainly, the aggregation of nanoparticles can influence the preservation of SAR values defined in Watts per gram as [6]:

SAR = C∆T/∆t

where C is the specific heat capacity and ∆T is the temperature rise during the time interval ∆t to deliver significant doses of heat with alternating magnetic fields well-tolerated by the normal tissues. Comparing SAR values of different magnetic nanoparticles requires much attention and care because they are affected by several parameters such as size, size distribution, chemical composition, and number concentration of the nanoparticles [59]. Magnetic modeling often evaluates the heating capacity of particles exposed to an alternating magnetic field through the product of hysteresis losses per cycle and the frequency of the external magnetic field. Such an approach reveals conflicting requirements. The so-called magnetic hyperthermia trilemma involves not only the type of particle used and its characteristics, such as volume, uniaxial magnetic anisotropy, and saturation magnetization, but also the amplitude and frequency of the alternating magnetic field and the sample concentration. Interaction between these parameters requires refined modeling and testing [60,61].

It is worth noting that single magnetic domain nanoparticles (namely, superparamagnetic), which tend to be less than 20 nm [62], can resonate within magnetic fields ranging from 10–10 MHz, which easily penetrate soft tissues and bones. Archetypal nanoformulations are iron oxide nanoparticles that are non-toxic, non-immunogenic, biocompatible, stable, biodegradable, and, to a certain degree, can be functionalized only to target tumor cells [63]. The safety of various iron oxide-based nanoparticles used as contrast agents in clinical use is well established [64].

Because of their benign specificities, nanosized iron oxide particles such as magnetite (Fe_3_O_4_), maghemite (γ-Fe_2_O_3_·Fe_3_O_4_), or hematite (γ-Fe_2_O_3_) are widely used in magnetic hyperthermia [65]. Iron oxide nanoparticles are far beyond the proof-of-concept stage with clinical benefits and applications as MRI contrast agents due to their optimal composition and biodistribution [2,66].

Finding effective SAR protocols for clinical conditions is challenging due to hyperthermia in both in vitro and in vivo scenarios. These require optimizing parameters, such as size, composition, and surface chemistry of magnetic nanoparticles, to achieve intracellular absorption, biodistribution, macrophage recognition, cytotoxicity, bioaccumulation drawbacks, and biodegradability [60,67,68]. *C. elegans* provides an ideal in vivo model for these purposes due to its information-rich and low-cost experiments. Nanoparticles’ chemistry and structure, coating, interactions with biological environments, and toxicity mechanisms must be understood to design biocompatible nanoparticles for diagnosis, therapy, and theranostics with a harmless toxicological profile [49].

The safety and biocompatibility of MNPs are paramount for diagnosis and therapy applications. These MNPs must be colloidally stable and resistant to corrosion and oxidation in aqueous and saline environments to be safely employed. Long-term stability and resistance to aging are essential for biomedical applications, along with the removal of generated by-products from biological tissue [52,69].

Some requirements must be considered to obtain high-quality magnetic nanoparticles for biomedical applications: excellent and persistent magnetic properties, and avoidance of nanoparticle aggregation. The coating material also plays an essential role in functionalizing MNPs with targeting molecules and drugs [70] and must be considered. These surface modifications can alter the nanoparticle’s toxicological profile and behavior. Gonzalez-Moragas and collaborators compared citrate and bovine serum albumin-coated MNPs in *C. elegans* and showed greater acute toxicity with citrate-coated MNPs [71]. The silica coating of Fe_3_O_4_ nanoparticles makes them suitable for drug conjugation and in vivo applications [55]. These studies in *C. elegans* reduce costs and synthesis time and minimize ethical issues before screening in mammalian models.

It is also possible to modify and engineer the surface of cells with polymers/nanoparticles to control the physiological parameters and the spatial distribution of modified cells. Minullina et al. have reported on the surface modification of *C. elegans* with polyelectrolyte multilayers and the direct magnetic functionalization of nematodes with biocompatible magnetic nanoparticles. The use of iron oxide MNPs functionalizing the nematode’s surface allows *C. elegans* to be manipulated by an external magnetic field [72]. The spatial manipulation, remote control of ion channels and neurons, and selective extraction of the nematodes can be important aspects of toxicity screenings and animal behavior studies, making the magnetic functionalization of nematodes a promising tool.

Model organisms, such as *C. elegans*, have been a thriving resource for studying magnetic field effects in various cells and tissues [73]. Invertebrate models such as *Drosophila melanogaster* have been used since the 1980s [74]. Interestingly, although the nematode *C. elegans* has been an emblematic model organism for studying the impact of a multitude of stimuli and environmental factors on behavior and physiology [75], only in the last two decades has it been used in studies related to the magnetic field. Ni [76], Cu [77], Cr [78], CeO_2_ [79], and Fe_16_N_2_ [67] are just some of the compositions that have been studied. It is important to highlight that magnetic nanoparticles can be found in soils, rivers, and the sea as residues from cosmetics, mines, industries, and cosmetics. Even when debris from large magnetic materials is released into the environment [80], it can suffer size fragmentation until it reaches nanometric size. Therefore, it is essential to study the toxicity of MNPs in vivo so they can be safely applied in different research areas.

Nanoparticle uptake by *C. elegans* nematodes has successfully assessed the toxicity of heavy metals and pollutants [81] and the interaction of nanoparticles in vivo [49]. Using MPNs in pollutant removal in wastewater leads to the possibility of subjecting *C. elegans* to contaminants exposure to evaluate parameters such as survival rate, growth, locomotion, and oxidative stress, among others [82]. The effects of internalized metal nanoparticles [83,84] on the nematodes’ bioaccumulation, reproduction, survival, and locomotion [67,85] were assessed. Furthermore, magnetic nanoparticles have been used to activate ion channels in *C. elegans* through heating [86]. However, only recently have internalized nanoparticles been used to increase the magnetic field in the nematode body locally and to study the subsequent impact on their metabolism [87].

Given the current technical difficulty of studying nanoparticles at nanometer resolution within living organisms, there is limited evidence for the mechanisms of internalization and translocation of nanoparticles in *C. elegans*. Several techniques have been applied to study the uptake and fate of nanoparticles in this animal model, including fluorescent microscopy [88], hyperspectral microscopy [89], and TEM [78], the most prevalent ones. Gubert et al. reported the first evidence of the intracellular location of Fe_16_N_2_ MNPs in *C. elegans*. They confirmed through TEM images that the MNPs could cross the cell membrane and be absorbed without causing animal death [67]. Although it should be verified by chemical identification or molecular mechanistic evidence, TEM can also provide clues about NP translocation routes, i.e., endocytosis [83].

It is important to emphasize that for some applications in nanomedicine (e.g., when used in treatments and as drug carriers), there is an advantage for MNPs to penetrate cells. When unabsorbed by cells, nanoparticles are excreted by *C. elegans*. Moreover, knowing the destination of MNPs within *C. elegans* is of paramount importance because, for example, when they are stored in the gonads of *C. elegans*, they can cause reprotoxicity, among other damages to the nematode [52,90].

## 5. *Caenorhabditis elegans* and Organic Nanoparticles

Organic nanoparticles have become part of strategies in biomedical product applications, such as human disease diagnosis, drug delivery, bioimaging, and cancer therapy. In particular, organic nanoparticles present easy processability, compatibility, and low toxicity, which are essential for application in biological systems [91]. Organic nanoparticles are present in several pharmaceutical products, favoring the bioavailability and effectiveness of water-insoluble agents, promoting the development of new drugs, and improving existing ones. The ability to form very small dispersions can turn insoluble materials into dissolved molecules by modifying their behavior without needing chemical agents with toxic potential [92].

Organic nanoparticles are solid particles of organic compounds such as polymers and/or lipids ranging from 10 nm to 1 µm. The technological development of nano-medicine has promoted the development of new materials and the refinement of existing ones, promoting the generation of more bio-tolerable organic nanoparticles, taking into account their non-persistence in environments [92]. In general, organic nanoparticles allow a better therapeutic approach due to their characteristics in the biological system, making them favorable and indispensable nowadays in the development of countless therapies for different human disorders.

In *C. elegans*, oral ingestion is the main form of organic nanoparticle absorption. Acute and chronic treatment regimens are applied in this model, in addition to topical exposures and microinjections. The animals’ feeding process is responsible for the absorption of organic nanoparticles since they feed on *Escherichia coli* (*E. coli* OP50) and consequently ingest the nanoparticles because they are widespread in food [39,93]. The repercussions at the biological level of exposure to organic nanoparticles are numerous, among which stand out changes in body size (evaluation parameter in developmental functions), brood size (reproductive success evaluation), pharyngeal pumping (points to the ingestion of food), in addition to body movement (indicating the neuronal viability and signaling performance).

### 5.1. Liposomes

Liposomes are self-assembled (phospho)lipid-based synthetic vesicles forming a bilayer (unilamellar) and/or a series of multiple bilayers forming a central aqueous compartment. Morphologically, they range in size from 30 nm to the micrometer scale, with a 4 to 5 nm phospholipid bilayer thickness [94]. Liposomes are used as delivery vehicles for drugs, proteins, nucleic acids, and contrast agents for bioimaging. They are used by different routes of administration, such as parenteral, pulmonary, oral, and transdermal, among others, to improve therapeutic efficacy and patient adherence to treatments [95,96].

The oral system is an essential route for the administration and absorption of nutrients in *C. elegans* and is also used for the administration of organic nanoparticles such as liposomes. The encapsulation of natural hydrophilic compounds is a tool used to decrease the solubility of the compounds in the growth medium where the animals are cultured. It is important in chronic exposure to maintain defined concentrations of the compounds (whose growth medium can absorb non-encapsulated compounds) and avoid possible interactions with *E. coli* OP50 as a food source [97].

Even then, the main aim of the papers discussed was on the compounds combined with the liposomes; here, we focused on liposomes’ benefits in *C. elegans*.

Liposomes have been successfully used in *C. elegans* to drive nutrient uptake in axenic media. Axenic medium is an alternative to provide nutrients and promote normal development in *C. elegans* independently of *E. coli* feeding. The liposomes mimicked the phospholipid bilayer found in the plasma membrane of the bacteria *E. coli* and were designed to have a size between 0.1 and 3 μm, which could be ingested by *C. elegans*. Despite presenting a lipid configuration, liposomes cannot replace the nutrient role of sterol, which needs to be offered as a nutrient. Altogether, the results pointed to liposomes as an effective tool to investigate the effect of various nutrients on the growth rate, with greater control over nutrient delivery [98].

The positively charged phosphatidylcholine liposomes (Lactyferrin Classic Drinkable Sesderma) were used as oral supplementation in *C. elegans*. As targeting biomedical applications, the phosphatidylcholine liposomes (size 80–150 nm) were designed for optimal delivery across the gastrointestinal and blood–brain barriers. The findings suggested that the nutraceutical product based on lactoferrin liposomes could improve the immune system and antioxidant capacity, extend lifespan, and reduce amyloid *β* peptide toxicity in *C. elegans* [99].

Besides nutrients drive, cationic liposomes were encapsulated with proteins of interest to be administered in *C. elegans* orally, which can avoid protein degradation in the gut and allow adsorption by body tissues. The authors used an antibody to inhibit α-synuclein aggregation and successfully rescue the disease phenotype in a *C. elegans* model of Parkinson’s disease [100].

Liposomes can orally deliver hydrophilic antioxidants (ascorbic acid, N-acetyl-cysteine, reduced glutathione, and thioproline) into the *C. elegans* gut. That increased lifespan compared to other delivery methods [101].

The use of liposomes as plant extract oral carriers also has been explored. The liposomes containing the extract of purple *pitanga* fruit (*Eugenia uniflora*—PPE) attenuate *C. elegans* total lipid and triacylglyceride levels in a high cholesterol protocol. The liposome use can retain encapsulated bioactive principles, prevent the fast elimination or degradation (e.g., by bacteria, light, environment conditions) before worms ingest, and facilitate estimating the concentration estimation due to the reduction in extract permeability through the nematode growth medium [97]. The encapsulation of betalains plant pigments in liposomes was administered in *C. elegans*. In this case, the liposome’s primary role was maintaining the bioactive properties of betalains, evidenced by increasing worms’ survival [102].

Liposomes were conjugated to carbon nanotubes and tested in *C. elegans*. A supramolecular nanohybrid based on liposomes and carbon nanotubes presented low cytotoxicity, high cell permeation, and the ability to absorb and convert NIR light into thermal energy. The last advantage, in particular, is useful to facilitate the controlled release of substrates [103]. The nanohybrids allowed the remote activation of amiloride-sensitive sodium channels in *C. elegans*, which could be observed through a vigorous movement forward after laser irradiation.

*C. elegans* can be used to validate the entrapment of antibiotics in lipid nanoparticles (LipNanoCar technology), with average sizes ranging from 100 to 300 nm. Antibiotics Cystic Fibrosis therapy in liposomes has been tested in the *C. elegans* model after bacterial infection [104].

Thus, liposomes are a very noteworthy compound carrier tool and a promising perspective to work around administration challenges in *C. elegans*.

### 5.2. Nanoemulsion

Nanoemulsions are heterogeneous formulations of two different immiscible liquids stabilized by surfactants. It is one of the most promising types of nanocarriers and can increase drug penetration by increasing its bioavailability. Physical-chemical stability, high load capacity of drugs inside, low toxicity, rapid release, and penetration of the drug are some of the characteristics that make this technology viable in the production and improvement of the activity of numerous drugs present in the market [105].

The nanoemulsions present droplet sizes on the nanometric scale, between 10 and 1000 nm, making them kinetically stable but thermodynamically unstable due to the system’s free energy compared to the separate phases. The stability of nanoemulsions is the central point for medical and biomedical applications, whose physicochemical changes directly impact the pharmacological properties of the formulation. The large surface area of nanoemulsions increases the absorption and effectiveness of nanoencapsulated drugs, in addition to protecting such drugs from different post-application biological systems, such as pH changes and enzymatic degradation [106].

The degree of saturation of the oily constituents used in the construction of nanoemulsions interferes with their characteristics, including their bioactivity. Nanoemulsions formed from an oil phase composed of olive oil, soybean oil, and linseed oil matrices induced some biochemical changes in the animals. When analyzing the treated animals, they did not show significant changes in body length or significant results in body width or pharyngeal pumping rate [107].

The nanoemulsions have a high-fat supply, and the excessive intake of fat-rich foods is directly related to lipid metabolism. When investigating the lipid-lowering effects of treatment with nanoemulsions, there were significant increases in the relative total triacylglycerol molecular content and its accumulations, with increases of 154.07%, 142.61%, and 109.30% in animals treated with olive oil, soybean oil, and linseed oil, respectively [106].

The antioxidant system of *C. elegans* is active, mainly due to the activity of the enzymes superoxide dismutase (SOD) and catalase (CAT), which control the damage caused by high levels of reactive oxygen species (ROS) [108]. SOD prevents cellular oxidation by converting O^2•−^ into H_2_O_2_, while CAT converts H_2_O_2_ into water and oxygen. The researchers reported that animals treated with nanoemulsions composed of olive oil, soybean oil, and linseed oil showed decreases in SOD and CAT activity when compared to the control group, showing that oily constituents in nanoemulsions with different saturations can affect (decrease/increase) the activity of antioxidant enzymes in the nematodes [106].

### 5.3. Dendrimers

Dendrimers are synthetic polymers characterized by repetitively branched units that emerge from a single point, with anionic, cationic, and neutral terminal functionalities exposed on their surface [109]. They are nanometric scale, globular, symmetrical, homogeneous, and monodisperse [110]. Dendrimers are used as drug and gene carrier systems, but some have therapeutic activities of their own because they have antifungal, antibacterial, and cytotoxic activities. Dendrimers are biodegradable, which may increase their applicability. It has high penetrability in biological tissues, including barriers and cell membranes, and is currently widely used as a gene delivery system within molecular biology [111].

*C. elegans* showed different behavioral modifications when exposed to Poly(amidoamine) dendrimers (PAMAM). Parameters for characterizing the size of the population were modified since animals treated with PAMAM for 48 and 72 h showed a decrease in the population number compared to the control [112]. At the transcriptome level, PAMAM modified the expression of 47 genes (29 upregulated and 18 suppressed) in 6 metabolic pathways. Among gene expression, pathways related to cytoplasmic ribosomal proteins, glycolysis, DNA replication, LIN-12-Notch lateral signaling, sex determination, and translation factors showed modifications (Table 2).

PAMAM G3 dendrimers are synthesized from an ethylenediamine core. *C. elegans* were exposed to PAMAM G3 for seven days at different concentrations, and animals treated with G3^2B12gh^ showed no significant differences in survival rate over the evaluated days compared to unexposed worms [113].

The number and nature of surface functional groups are the factors that determine the toxicity of dendrimers. Cationic dendrimers show high toxicity, while anionic and neutral ones show low or no toxic effects. Interactions between negative charges present in biological membranes and positive charges on the surface of dendrimers can explain the toxicity of cationic dendrimers. Such interaction causes the formation of nanopores in the cell membrane, promoting the leakage of cell contents and eventual cell death [114].

## 6. *Caenorhabditis elegans* and Carbon Nanosystems

Carbon nanotubes are concentric cylinders of graphite layers, frequently produced under chemical vapor deposition. Due to their remarkable properties, including chemical, electronic, mechanical, and optical, carbon nanotubes are often used in nanotechnology. Their wide versatility allows for significant advances in drug delivery, biosensors, molecular imaging, and diverse engineering [115].

The most widely used and studied carbon nanotubes are graphene, graphene oxide, fullerenes, and nanodiamonds. The functionalization of carbon nanotubes with strong acids and covalent attachment of organic groups on their surface makes them more suitable for applications in medicine [116]. These structures have applications in the health area and can be used as delivery systems for vaccines, drugs, or protein carriers. Carbon nanotubes can cross membranes and penetrate biological tissues, making them suitable for drug delivery. They have been studied as nanocapsules performing the “magic bullet” concept, where the drug is encapsulated and ejected once inside the cell [116,117].

Concerns about toxic environmental and human impacts go hand in hand with the broad possibilities of using carbon nanotubes, making extensive toxicity studies necessary to evaluate the acute and chronic toxic effects triggered by these particles [118]. Despite the high applicability, carbon nanotubes have a high chance of dissociation in biological fluids and can cause toxic effects on the organism [119]. This effect occurs due to changes in the shape and size, chances of impurity at the synthesis time, particle aggregation, final chemical composition of nanoparticles, and route of administration. In humans, carbon nanotubes can produce ROS, oxidative stress, inflammation, and damage to proteins and DNA, triggering actions such as apoptosis and enzyme inactivation. These nanoparticles are resilient to degradation in the environment and may persist for an unknown time [120].

In order to study such aspects, many authors presented *C. elegans* as a sensitive organism capable of responding to some nanomaterials and, therefore, could be considered a feasible biosensor for early monitoring of the presence of nanoparticles. This section intends to show the potential of *C. elegans* as a non-mammalian model for the biological evaluation of the toxicity and applications of carbon nanotubes. We explored the exposure regime and primary endpoints tested in the worm for fullerenes, graphene quantum dots, multiwalled carbon nanotubes (MWCNTs), single-walled carbon nanotubes (SWCNTs), and graphene oxide investigations. The main results discussed in the following sections were assembled together in Table 3.

### 6.1. Multiwalled Carbon Nanotubes

MWCNTs consist of concentric layers of graphene sheets rolled up, where smaller diameter tubes are encased in larger diameter ones. It has unique properties, such as small size, lightweight, large surface area, stability, rigidity, extraordinary tensile strength, and efficient heat conduction [121]. Given this, MWCNTs have presented great potential in biomedical and pharmaceutical applications, including drug and gene delivery, biosensors, and tissue engineering [122].

In the wild type, MWCNTs functionalized with a hydroxyl (OH-MWCNTs) with a small external diameter (8–15 nm) were more toxic than those with a large size, and those with a shorter tube length (0.5–2 μm) exhibited more significant toxicity than those with a longer tube length. In *pmk-1* and *cep-1(138*) mutant strains, short-MWCNTs induced lower survival when compared to the wild-type strain, and long-MWCNTs promoted changes in the survival of different mutant strains, indicating that a change in tube length can promote toxicity through different mechanisms of action [123].

Using worms of the *zaEx5* [*let-7*::GFP] strain, *let-7*::GFP miRNA expression and its *hbl-1* and *lin-41* targets were studied. After exposure to MWCNTs at a concentration of 10 μg L^−1^, it was observed that there was a reduction of miRNA in the nematode after exposure. Consequently, there was an increase in the expression of HBL-1 and LIN-41. Thus, it was hypothesized that exposure to MWCNTs can disrupt the worm’s development, according to the expression of *let-7* and the targets [124].

In the *C. elegans* N2 strain, exposure to MWCNTs did not significantly affect worm survival and body length after 72 h of exposure on plates (10^10^ particles mL^−1^). However, MWCNTs changed the expression of 16 genes (9 up-regulated and 7 down-regulated) representing 7 metabolic pathways, including aging, immune responses in the intestine, insulin signaling pathway, dauer formation, and metabolic pathways [112].

Some microRNAs (miRNAs) can be dysregulated in response to MWCNTs. A complete analysis of the transcriptome showed a change in 342 mRNAs expressed after exposure to MWCNT in 0.1 μg L^−1^, 149 were up-regulated, and 193 were down-regulated, 89 of which were potential targets of *mir-35* [125]. There is an increase in *mir-35* expression after exposure to MWCNTs, which induces an intestinal protective response. Moreover, MAB-3 was identified as a target and acted upstream of DAF-16 [126].

The adverse effects of MWCNT depend upon surface functionalization as well as the model organisms tested. The authors evaluated the toxicity following exposure to pristine as well as surface-functionalized MWCNTs following hydroxylation-oxygenation (O^+^), amination (NH_2_), or carboxylation (COOH) of carbon nanotubes. No significant impacts on *C. elegans* wild-type (N2) survival were detected after 24 h of exposure to all four MWCNTs. Although a significant reduction in reproduction potential was found in pristine MWCNT-exposed worms [127].

Through genetic sequencing techniques, the role of *mir-355* in the toxicity of MWCNTs was studied, influencing the functions of insulin signaling pathways and elaborating an altered mRNAs–miRNAs network. As a result, some genes involved in the insulin signaling pathway were detected, in addition to an increase in the percentage of DAF-16::GFP. Furthermore, the *daf-2* gene, the initiator of the insulin signaling pathway in nematodes, may have its functions suppressed, acting as a target gene for *mir-355* [128].

Changes in lifespan, motility, and reproductive behavior parameters were analyzed using acid-functionalized MWCNTs (fMWNTs). There were no significant changes in the lifespan of the worm. However, after the tenth day, the motility of those animals exposed to a concentration of 100 mg L^−1^ and 500 mg L^−1^ was reduced. fMWCNTs were detected in the offspring generated by the worms exposed at L4, and the spawning rate of a single worm decreased by 12% in those exposed to a concentration of 500 mg L^−1^, thus deducing that these particles cause a generational accumulation in *C. elegans* and can reduce the future population [129].

### 6.2. Single-Walled Carbon Nanotubes

SWCNTs are single-layer graphite structures rolled into a tube with hydrophobic characteristics and a diameter ranging from 1 to 2 nm. They can be used for industrial, biomedical, and environmental purposes. That comprises SWCNT usage for drug delivery, bioimaging, sensors, nanoelectrodes, batteries, supercapacitors, electron field emitters, catalysts, polymer additives, and reinforcements in nanocomposites [130,131].

SWCNTs modified with an amide group (α-SWCNTs) to facilitate their solubility and absorption were analyzed in *C. elegans* to verify their toxic effects. When exposed to a concentration of 500 μg mL^−1^, a lifespan reduction in the reproduction and expression of the BOW phenotype in the gonads was observed, causing the eggs to be hatched still inside the animal’s body, indicating compromised egg-laying. Moreover, through genomic analysis, the positive regulation of 14 genes was verified, but 2085 genes were negatively regulated after exposure [132].

SWCNTs−COOH have been tested in *C. elegans* to verify the effects on lethality, lifespan, growth, reproduction, locomotion, ROS generation, and the antioxidant system. SWCNTs−COOH could affect lifespan, growth, reproduction, and locomotion behavior. The nematodes showed ROS generation increasing, followed by changes in the expression of antioxidant genes such as *sod-3*, *ctl-2*, and *cyp-35A2* at low doses of SWCNTs−COOH [120]. 

To determine the biocompatibility of the Cys-SWNTs, in vivo toxicity studies were performed on N2 wild-type *C. elegans*. It was observed that the cysteine-functionalized SWNT platform can be successfully synthesized using a straightforward procedure and does not exhibit signs of acute or chronic toxicity in the *C. elegans* model organism. Specifically, worms exposed to these nanotubes did not exhibit adverse physiological effects in the short-term, long-term, or reproduction viability. These results are a promising indication that Cys–SWNTs may be safe in larger organisms and may be further scrutinized for potential applications in biomedicine [133].

On the other hand, NIR fluorescent single-walled carbon nanotubes (SWCNTs) have been explored for in vivo imaging within the *C. elegans* gut. The SWCNTs are biocompatible and do not affect the worms’ viability or reproduction ability (0.1 to 300 mg L^−1^) [134].

### 6.3. Fullerenes and Graphene Quantum Dots

Fullerenes (buckyballs) are a class of carbon allotropes with a spherical or ellipsoidal shape. Their size depends on the number of carbons—C60, C70, C80, and more. The most common fullerene structure, C60, consists of 60 carbon atoms arranged as 20 regular hexagons and 12 regular pentagons. The insolubility of pure fullerenes in water turns out to be an issue for applications in biomedical fields. The functionalization of groups such as –COOH and –OH is used to improve the dissolution of fullerenes. However, the water-soluble fullerenes tend to clump together rather than remain separate molecules [135]. 

Studies were carried out using *C. elegans* to evaluate the possible toxic effects of hydroxylated fullerene (fullerol) nanoparticles, considering lifespan, size, and reproductive behavior. At a concentration of 100 µg mL^−1^, the lifespan of worms at the L4 larval stage rapidly decayed, with a 50% survival rate by day 5 of adulthood. As well as the lifespan, the number of eggs laid by the animal was also reduced, and its body size. Large numbers of undigested bacteria point to an intestinal disorder induced by exposure. Moreover, using the mutant strains in the *ced-3* and *ced-4* genes, it is revealed that the nanoparticles induce the apoptotic process in the worm [136].

However, when exposing the nematodes to concentrations between 0.01 and 100 µM of polyhydroxylated fullerene (fullerenol), there is no induction of adverse effects on the survival or growth of the animal. The TK22 strain, a *mev-1* mutant sensitive to oxidative stress and carrier of an accelerated aging behavior, is used to investigate intestinal autofluorescence, which is used as a biomarker of worm aging through the accumulation or not of lipofuscin. It was found that fullerenol at specific concentrations can reduce autofluorescence in these mutants, providing data that support the use of fullerenol as a contributor to delaying aging [137].

Graphene quantum dots are quasi-spherical nanoparticles produced by destroying more extensive carbon materials, such as carbon nanotubes, graphene, graphene oxide sheets, or carbon fibers, by strong acid oxidation, hydrothermal or solvothermal treatment, etc. Graphene quantum dots have remarkable photochemical and photoluminescent properties, are dissolved in most polar solvents without additional chemical treatments, and have excellent stability compared to other fluorescent dyes, making graphene quantum dots suitable for use in bioimaging [90].

In *C. elegans* exposed to nitrogen-doped graphene quantum dots (N-GQD) at concentrations ranging from 0.1 to 100 mg L^−1^, the animal’s essential functions were maintained, with no changes in survival, life expectancy, or intestinal production from ROS. In animals with mutations in the *sod-2* or *sod-3* genes, no evidence of lethality was observed, and prolonged exposure to the highest concentration (100 mg L^−1^) did not significantly affect the expression patterns of genes encoding SOD in exposed worms and their F1 progeny. There were also no changes in the expression patterns of the genes *mev-1*, *isp-1*, *gas-1*, *clk-1*, *ctl-1*, *ctl-2*, and *ectl-3*, these results being essential to increasing the data about the toxicity of N-GQD in vivo [138].

Due to the autofluorescence of N-GQD and the transparency of the worm’s body, it is possible to analyze the accumulation of the nanomaterial in a given region. In worms exposed to 200 μg mL^−1^ of N-GQDs for 24 h, it is possible to observe an agglomeration in the animal’s intestinal system. In those treated for 48 h, the material was accumulated throughout the worm’s entire length, indicating possible toxicity. Changes in locomotion were also induced through exposure, which is a parameter for assessing neurotoxicity. In addition, there is a significant reduction in the expression of the *dat-1* and *eat-4* genes, indicating more significant neurotoxicity against dopaminergic and glutamatergic neurons [139].

### 6.4. Graphene Oxide

Differently from MWCNT and SWCNT, graphene presents a two-dimensional (2D) shape with a monolayer where carbon atoms are arranged in a honeycomb lattice structure. Graphene has considerable potential for applications due to its properties, including high thermal and electrical conductivity and stability, high flexibility and elasticity, hardness and resistance, and large surface area [140].

The mechanism to counteract graphene oxide toxicity by worms involves the activation of some miRNA, including *mir-60*. *miRNAs* are short noncoding RNAs that can inhibit gene expression post-transcriptionally. *mir-60* has a protective role in the regulation of apoptosis in *C. elegans* that triggers a reduction in the reprotoxic effect caused by graphene oxide. One of the possible targets of *mir-360* is CEP-1, an ortholog of human tumor suppressor p53, which is involved in suppressing the DNA damage-apoptosis signaling cascade [141]. Those mechanisms are tightly related to the epigenetic responses in *C. elegans* against graphene oxide [141].

In addition, the antioxidant N-Acetyl-L-cysteine (NAC) effectively suppressed graphene oxide’s toxicity with increased oxidative stress resistance. Worms with RNAi-induced antioxidative genes *sod-1*, *sod-2*, *sod-3*, and *sod-4* knockdown were more sensitive to graphene oxide. 3-MA increased the expression of SOD-3 under graphene oxide exposure conditions and exacerbated the toxicity of graphene oxide under the anti-oxidation inaction condition by *sod-3* RNAi. In contrast, NAC reduced autophagy levels in graphene oxide-exposed nematodes and increased tolerance to graphene oxide in autophagy-defective worms. These results suggested that autophagy and the antioxidative response provide complementary protection against graphene oxide in *C. elegans* [142].

Furthermore, graphene oxide-induced autophagy was induced in *C. elegans* by increasing the LGG-1::GFP puncta (10 mg L^−1^ to 100 mg L^−1^). Graphene oxide-induced autophagy was increased under the inhibitor 3-methyladenine (3-MA), silencing the autophagy genes *lgg-1*, *bec-1*, and *unc-51*, whereas the autophagy activator rapamycin could reduce it. The antioxidant system seems to be induced against graphene oxide toxicity since the antioxidant NAC effectively increases resistance to oxidative stress. Worms under antioxidative genes *sod-1*, *sod-2*, *sod-3*, and *sod-4* knockdown were more sensitive to graphene oxide [142].

Graphene oxide uptake could be detected with confocal Raman spectroscopy in *C. elegans* after 2 h and accumulated around the reproductive system at 48 h of exposure. This was followed by reprotoxicity, fat metabolism, and increased oxidative stress [143]. Exposure at a concentration of 100 mg L^−1^ could reduce the body length and lifespan, and 0.1 to 100 mg L^−1^ did not induce the lethality of nematodes. Moreover, graphene oxide exposure at concentrations of more than 1 mg L^−1^ led to reproductive toxicity by decreasing the brood size in nematodes [85].

The prolonged exposure to thiolated graphene oxide at low concentrations (100 μg L^−1^) resulted in toxicity in the intestine, neuronal system, and reproductive organs. Thiolated graphene oxide decreased the expression of *gas-1,* which encodes a subunit of mitochondrial complex I. On the other hand, the *gas-1* mutant presented toxic effects (10 μg L^−1^) and severe thiolated graphene oxide accumulation in the body of *C. elegans* [144].

Moreover, exposure to graphene oxide alters the worm’s lipid metabolism and parameters such as reproduction, impairing spermatogenesis in the wild strain and the four fat-related mutants, *fat-5*, *fat-6*, *fat-7*, and *nhr-49*. At 10 mg L^−1^, graphene oxide exposure resulted in a disturbance in fatty acid metabolism due to the suppression of gene expression of genes associated with these acids. Developmental delay and egg position defects are also in *fat-6* and *fat-7* strain worms [143].

**Table 3:** Impacts under different carbon nanosystems exposition in *Caenorhabditis elegans*.

Multiwalled Carbon Nanotubes (MWCNT)				
Concentrations/Time	Exposure Conditions	Endpoint	Effects	Reference
100 mg L^−1^ for 24 h 50 mg L^−1^ for 72 h	K-medium *E. coli* OP50 Young adults (day-3)	Survival	No changes	[127]
		Reproduction	Decrease (only pristine coated)	
100–500 mg L^−1^ for 3–15 days	NMG medium surface (5.203 mg L^−1^ FUDR, *E. coli* OP50). L4 until death	Survival	No changes	[129]
		Movement	Decreased (100 mg L^−1^, 500 mg L^−1^)	
0.1 μg L^−1^	NMG medium surface *E. coli* OP50 L1-Day-1 Adult	Gene regulation	149 genes up-regulated and 193 genes down-regulated	[125]
10^10^ particles per mL for 72 h	NMG medium surface *E. coli* OP50 L1-Day-1 Adult	Transcriptome	Upregulated *daf-2* (3.18) *daf-16* (1.01) *bet-1* (2.48) *rpl-33* (1.01) *rps-14* (1.01) *rps-20* (1.01) *par-1* (1.01) *lin-45* (1.01) *lag-1* (1.01) *dnj-10* (2.12) Gene suppression *smk-1 *(−2.13) *rpl-31* (−6.50) *ubq-2* (−2.08) *daf-12* (−5.15) *spp-1* (−2.44) *hsp-60* (−2.17)	[112]
		Survival	No changes	
		Body length	No changes	
Single-walled carbon nanotubes (SWCNTs) SWCNTs−COOH				
Concentrations/Time	Exposure conditions	Endpoint	Effects	Reference
0.001 to 1000 μg L^−1^ SWCNTs− COOH for 24 h (acute exposure)	NMG medium surface L3/young L4	Lethality	No changes	[120]
		Lifespan	Decrease	
		Growth	Decrease	
		Reproduction	Decrease	
		Locomotion	Decrease	
		ROS	Increase	
		Antioxidant system	Increase the expression of *sod-3*, *ctl-2*, and *cyp-35A2*	
0.1–300 mg L^−1^ SWCNT and ssDNA-SWCNT for 4–24 h.	NMG medium surface or vials (NaCl 0.9%). Day-1 Adult	Reproduction	No changes	[134]
		Survival	No changes	
		Imaging acquisition	Fluorescent bio-imaging	
50, 100, and 250 mg mL^−1^ Cys–SWNTs for 3 h	NMG medium surface Suspensions of Cys–SWNTs in M9 buffer L4	Survival	No changes	[133]
		Lifespan	No changes	
		Brood-size	No changes	
100, 250, and 500 mg mL^−1^ a-SWCNTs for 48 h	NMG medium surface L1-Day-1 Adult	Survival	No changes	[132]
		Lifespan	Decrease	
		Reproduction	Decrease	
		Expression of the BOW phenotype	Activated	
Fullerenes and Graphene quantum dots				
Concentrations/Time	Exposure conditions	Endpoint	Effects	Reference
100 µg mL^−1^ hydroxylated fullerene (fullerol)	NMG medium surface *E. coli* OP50 L4- Day-5 Adult	Lifespan	Decrease	[136]
		Eggs laid	Decrease	
		Body size	Decrease	
		Apoptotic process	Increase	
0.01 and 100 µM polyhydroxylated fullerene (fullerenol) for 24 h	NMG medium surface *E. coli* OP50 L1	Autofluorescence	Decrease	[137]
200 μg mL^−1^ N-GQD for 24 h	K medium L4-Day-1 Adult	Neurotoxicity	Increase	[139]
Graphene Oxide				
Concentrations/Time	Exposure conditions	Endpoint	Effects	Reference
100 mg L^−1^ >1 mg L^−1^ for 24 h	NMG medium surface *E. coli* OP50 L4	Body length	Decrease	[85]
		Lifespan	Decrease	
		Brood size	Increase	
1–100 mg L^−1^ for 48 h	NGM medium surface *E. coli* OP50 L1- young adults	Survival	No changes	[141]
		Expression pattern of genes encoding p38 MAPK signaling	Increase *pmk-1*, *sek-1*, and *nsy-1* Decrease PMK-1::GFP expression in *sek-1(ag1)* or *nsy-1(ag3)* mutants	
>100 μg L^−1^ Thiolated graphene oxide	K-medium *E. coli* OP50 L1- Day-1 Adult	Reproduction	Decrease	[144]
		Locomotion	Decrease	
		ROS production	Increase	
		Intestinal permeability	Increase	
10 mg L^−1^ for 48 h	NMG medium surface K-medium L1- L4 larval stage	Uptake	at 2 h	[143]
		Bioaccumulation	at 48 h (reproductive system)	
		Reproduction	Impairment, suppression of spermatogenesis	
		Oxidative stress	Increase	
		Fat accumulation	Increase	
10–100 mg L^−1 ^ for 48 h	NMG medium surface *E. coli* OP50 L1–L4 larval stage	Autophagy	Increase	[142]

Blue: upregulated. Red: downregulated.

## 7. *Caenorhabditis elegans* and Nanopesticides

For years, chemical formulations have been the primary strategy for controlling agricultural pests and increasing production while protecting crops. Everyday adversities, such as outbreaks of insects, fungi, diseases, and weeds, have driven the widespread application of pesticides and their consequent presence in the environment. Despite their cost-effectiveness advantages, the immediate release of their active ingredients into the soil causes the loss of these products through degradation processes caused by light, rain, and microbiota metabolism, for instance. Therefore, there is a reduced delivery to the target, impacting their biological effectiveness and requiring several reapplications. This indiscriminate use also triggers various adverse environmental effects directly associated with a wide range of pollutants and micropollutants through their bioaccumulation, bioconcentration, and biomagnification [145].

Consequently, pesticides in water, soil, and food are also responsible for causing damage to non-target organisms (including invertebrates and vertebrates), selecting resistant species, and triggering outbreaks of secondary pests, directly and indirectly harming human health. Over the years, the demand for more sustainable and eco-friendly agriculture has been increasing. The pressure exerted on agrifood systems to avoid pesticides, fungicides, herbicides, and chemical fertilizers has stimulated the search for alternative methodologies with low cost and high benefits that aim to guarantee growth and greater production yields, at the same time, minimize the impact caused by pesticides to the environment and the society [146].

Nanotechnology has also specialized in providing green methods to increase agricultural production. Nanomaterials can be used in agriculture as fertilizers and pesticides (e.g., herbicides and fungicides). They are classified based on their chemical nature and may be organic or inorganic. Most formulations for nanopesticides are based on polymers, nanomaterials, and nanoemulsions [147]. The nanostructure protects the internal content (pesticide) and provides features such as slow, controlled, and sustained active ingredient release, increasing the product shelf life and improving the solubility of these molecules in water [145]. In addition, nanoencapsulation also allows using less toxic alternative formulations that are more sensitive, protect from environmental degradation, and control pathogens, weeds, and insects in crops. Those factors highlight the importance of using nanotechnology in agriculture and the food industry, reducing the use of harmful chemicals, and ensuring the safety and development of plantations. Another improvement has been made by using biopolymers instead of synthetic polymers as coating materials [148]. This has attracted attention due to their ecological nature and biodegradable character, as most biopolymeric materials are derived from plants and animals. Another eco-friendly approach promoted by nanotechnology is the synthesis of nanoparticles using plant extracts as reducing agents or as active ingredients due to their insecticidal and antifungal properties [149].

However, despite this new technology bringing alternatives and significantly improving the application of pesticides in agriculture, more is needed to know about the impacts of the nanopesticides on the environment and their safety to surrounding organisms [149,150]. Because it is at the nanoscale, the physical properties can change. Characteristics such as particle size, surface area, shape, and coating interfere with the nanoparticles’ cytotoxicity and, depending on the material, can lead to unexpected chemical and biological reactivity, causing higher toxicity to animals and humans. In vivo tests tend to be carried out on target organisms. However, it must be taken into account that only about 1% of all insects are considered pests, thus highlighting the need to exhaustively test the safety of nanomaterials in non-target organisms. Due to ethical issues and operational costs, the use of mammals for in vivo acute toxicity tests has been restricted, opening space for the benefit of simpler and alternative experimental models such as zebrafish, fruit flies, and, more recently, nematodes [93,150].

Besides *Eisenia fetida*, *C. elegans* is also considered a good in vivo organism for developing toxicological analyses. It is often used for nanoparticle studies since it is regarded as a non-target organism in the soil [151]. As this is a recent field of study, there are only three studies evaluating the safety of nanopesticides using *C. elegans* as an experimental model. The first study sought to evaluate the safety of three nanopesticides. The polymers used were chitosan tripolyphosphate, a biological polymer, solid lipid nanoparticles, and poly-epsilon caprolactone, a synthetic polymer. Unloaded and pesticide-loaded nanoparticles were used. However, when assessing the survival rate, the authors observed that all nanoformulations reduced the survival rate of the treated worms, including the unloaded nanoformulations, indicating that the nanomaterials presented toxicity for the nematodes and not the pesticides. In other assays, such as body size and brood size, it was observed that worms exposed to chitosan/TPP nanoformulations loaded or not with paraquat did not exhibit changes in these parameters.

On the other hand, the nanoformulations containing synthetic polymers significantly altered the body size, as well as the viability of the nematode eggs [152]. This study was not the first to detect that some materials used in the production of nanoparticles can be toxic to worms and other species [153,154]. Notably, chitosan/TPP were the less toxic nanoparticles, and therefore, that was a clue that biopolymers may be the best option for eco-friendly nanopesticides.

Therefore, the same group further investigated using another biopolymer, zein. This polymer is obtained from corn (*Zea mays*), and this study was used to coat nanoparticles loaded with neem oil. Neem oil is extracted from the Indian Neem tree (*Azadirachta indica Juss.*). It contains over 300 biologically active compounds, making it effective against many pests and acting as a fertilizer for agricultural production [155]. Due to its systemic and transmembrane activity, neem oil has a broad spectrum of action, inhibiting feeding, disrupting development, suppressing fertility and reproduction, and reducing ecdysone [156]. However, its short persistence time in the environment makes its use limited. The toxicity tests carried out in this study demonstrated that zein nanoparticles loaded or not with neem oil did not change the parameters of survival, reproduction, and body length in any of the tested concentrations. Notably, unloaded zein nanoparticles were safe for the worms, even after 48 h of continuous exposure. Other parameters tested showed that free neem oil decreased pharyngeal pumping and reduced GST-4 expression.

On the other hand, zein nanoparticles loaded with this oil did not affect these parameters. Pharyngeal pumping indicates whether the nematode is healthy, and its reduction can lead to food restriction. GST-4 levels are involved in detoxification and cell defense, and reduced levels can lead to oxidative stress and cell death. The results of this study suggest that the formulation of zein nanoparticles reduced the toxicity of the oil, indicating that natural biopolymers may be more compatible with non-target organisms [157].

Due to these promising results indicating that zein is a good strategy for delivering the nanopesticide, the same group evaluated the toxicity of zein nanoparticles loaded with clove oil and its main bioactive compound, eugenol, which are both highly volatile oils. The survival tests showed that the free clove oil did not show toxicity. However, the nanoformulation containing the oil did, contrary to what was observed in the survival tests with eugenol, in which toxicity was reduced in the nanoformulation. Nanoparticles and free oils reduced the reproduction rate, whereas essential oils decreased pharyngeal pumping, while nanoformulations did not. This result may be associated with the chemosensory system of the worm, which slows down the pharyngeal beat in the presence of odorous substances, which may be harmful. The nanoformulations reduce the odorant effect of the oils, so the pharyngeal pumping is not altered. The exposures did not change the expression of GST-4 and the antioxidant SOD-3, nor the number of apoptotic bodies, evidencing the safety of nanoformulations for non-target organisms [158]. Finally, the study showed that the nanoencapsulated forms of clove oil and eugenol present insecticide activity against *Drosophila melanogaster*, indicating the efficacy of these products as bioinsecticides.

Although the nematode is considered a good in vivo model for toxicological analyses, further studies are still needed to fully assess the effects of nanomaterials on the worm’s already elucidated pathways.

## 8. *Caenorhabditis elegans* and Nanoplastics

### 8.1. Nanoplastic Definition

Nanoplastics are synthetic polymers with dimensions ranging from 1 nm to 1 μm and can exhibit colloidal behavior [159,160].

Despite having “nano” in the name, nanoplastics cannot be compared with other nanomaterials. The main differences are related to the production pathways, the main objective of production, and physical and chemical properties. Regarding the aim of production, a nanomaterial is “intentionally produced for commercial purposes to have specific properties or specific composition” [161]. This definition cannot be transferred to nanoplastics since it is not manufactured in “nano” size, and most of the nanoplastic in the environment originated from the degradation of macro or microplastic items [162].

Mapping nanoplastic sources is not an easy task. Plastic degradation in the environment can lead to different nanoplastic characteristics. The plastic surface and structure in the environment are uncontrolled and shaped by physical and chemical parameters (e.g., UV radiation intensity, pH, salinity, soil or sand composition/granulometry, and organic matter). Commercial plastics generally have additives to give physical/chemical properties required for commercial purposes (color, flexibility or hardness, resistance to solar irradiation, and bacterial or fungal attacks, among others) [160]. In the degradation process, additives can be released into the environment, and the colloidal behavior of nanoplastics can aggregate toxins such as pesticides.

### 8.2. Nanoplastics and the Risks to Human Health

Plastic products are becoming a global environmental problem due to their large-scale production, which currently exceeds about 300 million tons, and the lack of waste treatment. Over time, these plastics undergo biodegradation processes and lose their integrity. The gradual decomposition can result in microplastics (<5 mm) and nanoplastics (<1 μm). Among these plastics, polystyrene, and polyethylene are examples [163,164].

There are two critical characteristics of nanoplastics. The ability to adsorb toxic molecules, serve as a carrier, and be uptaken by cell organisms, and cross the blood–brain barrier [165]. Ingestion, inhalation, and dermal contact are the three most common routes of nanoplastic contamination. When ingested, the particles can reach the gastrointestinal system, and their accumulation can trigger an inflammatory reaction [166]. Inhalation can occur in situations such as the abrasion of plastic surfaces and the suspension of these particles in the air. Some studies even consider the probability of nanoplastics crossing the dermal barrier and resulting in oxidative stress processes in human epithelial cells [167].

In the human food chain, nanoplastics have been detected in foods such as honey, beer, fish, seafood, and sugar. During the analysis of human feces, plastic residues were found, which added even more emphasis to the premise of the presence and ingestion of nanoplastics in human life routines [168]. Due to their small size, nanoplastics can quickly be deposited in the lung alveoli and travel to any part of the body through the circulatory system, an example of an inhalation route of contamination in the human body [169,170].

In 2015, a paper was published describing, through an ex vivo model, the ability of polystyrene nanoparticles to transfer through placental perfusion. These findings have shown the importance of further studies on the interaction of nanoplastics and their harmful actions in the human body [171]. Other studies using models such as *C. elegans* are needed in this context to identify routes and possible effects on human health [167].

### 8.3. Lack of Studies Using Environmental Nanomaterial

The scientific community needs to improve methods for finding and measuring nanoplastics in natural water and soil samples. Technology is improving for identifying polymer signatures in seawater. However, detecting nanoplastics in the environment and in organisms is problematic since only plastic combinations can be identified, not specific polymers.

However, dark-field hyperspectral microscopy has been tested to identify synthetic micro- and nanoplastics (up to 100 nm) using *C. elegans* as a model to demonstrate microplastic ingestion and tissue distribution [140], as these nematodes are optically transparent. There is still a gap in the literature as studies rarely analyze environmental samples.

An alternative to performing toxicological studies and/or analyzing plastic degradation is to produce nanoplastic artificially in the laboratory from macro- or microplastic. It is possible to identify some studies aiming to produce nanoplastic from items such as polystyrene from disposable coffee cup lids [172] and fragmentation of expanded polystyrene exposed to sunlight [173].

Some key points that are necessary to take into account when dealing with environmental plastic samples are: Are nanoplastics still associated with additives? Are they associated with organic pollutants? Or heavy metals? It is necessary to develop analytical methods adapted to characterize the physical properties and the chemical composition of these nanoplastics and to study the evolutions regarding the aging of plastic debris within the continuum of size categories (from macroscopic to micrometric down to the nanoscale).

It is rare to find scientific literature using plastic marine debris collected in the environment to produce nanoplastic [174]. This is a gap because the environment will determine the characteristics of the nanoplastic. A nano of soil will not have the same features as a nano of freshwater or marine environments. Moreover, the dispersion of additives and the adsorption of contaminants in the water are probably higher than in the soil [175].

### 8.4. Studying Nanoplastic Toxicity Using Caenorhabditis elegans

Studies using laboratory-produced nanoplastics have already tested the ecotoxicity of nanoplastic for soil organisms such as *C. elegans*, identifying energy budget impairment and decreases in reproductive fitness [176] and testing *C. elegans* nanoplastic laboratory-produced exposure at predicted environmental concentrations [177,178]. 

Oral intake remains the main route of absorption of nanomaterials, including nanoplastics. In a laboratory setting, nanomaterials were applied to worms, and some endpoints, including premature aging, reduced health and longevity, behavioral impacts, conserved signaling pathways (e.g., oxidative stress), neurodegeneration, and reprotoxicity, were assessed [179].

Smaller particles may be harmful because they can be easily ingested, reducing food intake. Some studies have linked the toxic effects of nanoparticles on *C. elegans* reproduction, but these effects can also occur from injury to the food, not just the particles. A high density of bacteria can protect against toxicity [179]. To avoid experimental errors, the number of bacteria should be constant in experiments.

**Figure 1:**
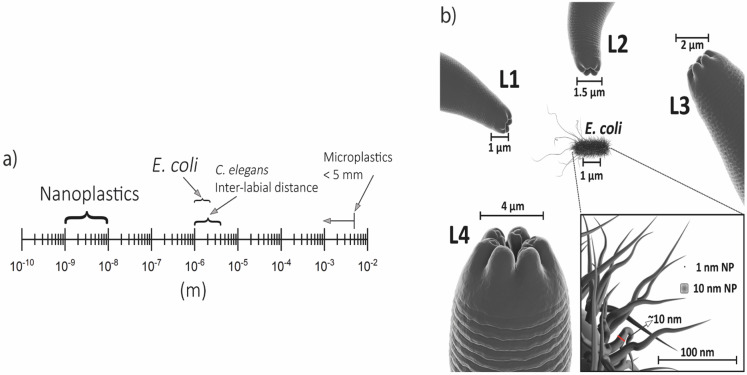
3D image comparison between the size of *C. elegans* mouth in different larval stages, *E. coli* bacteria, and nanoplastic (NP). (**a**) Scale comparing the sizes of micro- and nanoplastics, *E. coli* bacteria, and *C. elegans* (L1: 1 μm; L2: 1.5 μm; L3: 2 μm; L4: 4 μm). (**b**) 3D representation of the organisms and nanoplastics. This illustration was created in Blender (Community, B. O. (2018). Blender—a 3D modeling and rendering package. Stichting Blender Foundation, Amsterdam. Retrieved from http://www.blender.org (accessed on 20 February 2023)).

Observing the nanoparticle size and *C. elegans* larval stage capability to ingest is crucial for the experiment’s success (Figure 1). The size of *C. elegans* mouth, as expected, increases through the larval stages (L1: 1 μm; L2: 1.5 μm; L3: 2 μm; L4: 4 μm). Compared to the size of *E. coli* (1 μm), worms can easily consume the nanoplastics. However, whether the experiment is performed with microplastics (<5 mm), the size is an issue for oral exposure. Even then, toxic effects were observed with microplastic in *C. elegans*, which could occur by limiting food availability or causing hypodermis damage. Smaller particle sizes may be more toxic due to more extended periods of action, staying in the gut for longer, or easier and preferential ingestion [180].

Overall, studies using nanoplastics and *C. elegans* are promising fields of study, especially considering that nanoplastics are emerging contaminants that can adsorb other environmental pollutants, such as additives and pesticides.

#### Nanopolystyrene and *Caenorhabditis elegans*

Beyond all the nanoplastic types, the most explored until now in *C. elegans* is undoubtedly the nanopolystyrene. Polystyrene, the polymer associated with nanopolystyrene sources, is one of the most widely used polymers. This polymer may be used in toys, toothbrushes, cup lids, and styrofoam manufacturing processes [181,182]. Once exposed at high concentrations (100 and 1000 μg L^−1^), nanopolystyrene could reduce *C. elegans* lifespan, induce severe intestinal ROS production, and decrease locomotion behavior during aging. Besides, it caused suppression in the mitochondrial unfolded protein response (mt UPR) and the innate immune response by reducing the expression of HSP-6 and Mn-SOD encoding genes [183]. When chronic exposure to nanopolystyrene occurred, the autophagy process was activated as a protective response mechanism for environmental toxicity [184].

*C. elegans* is presented as a model to study circadian rhythms that can be evaluated by changes in behavior, metabolism, protein activity, and transcriptional regulation [185]. Nanopolystyrene can disturb circadian rhythms in *C. elegans* through ASH neurons and G protein-coupled receptor kinase (GRK2) signaling in the chemotaxis response. Besides, ROS production induced by nanopolystyrene exposure and peroxiredoxin-2 (PRDX-2) is mainly related to changes in circadian rhythms. That triggers the stress resistance reduction, in which transcription DAF-16/FOXO modulates signaling [186]. 

Moreover, the nanopolystyrene (100 nm, at 0.1–10 mg L^−1^) could affect *C. elegans* proteome dynamics. After 48 h of exposure, 136 out of 1684 proteins evaluated were differentially expressed, and 108 of these proteins were upregulated. Such *C. elegans* proteins have been related to translation, ribosome biogenesis, proteolysis, kinases, protein processing in the endoplasmic reticulum, and energy metabolism. Remarkably, the changes in proteome followed by nanopolystyrene exposure are corroborated by the phenotypic impacts observed in *C. elegans* [187].

The aim of neuroregulation under nanoplastic toxicity was also investigated. When chronically exposed (L1-adult day-1 or 72 h) to nanopolystyrene (25, 50, and 100 nm), neurodevelopmental toxicity was observed, followed by ROS production and mitochondrial dysfunction. This inhibited movement (body length, head thrashes, body bends) and dopamine contents [188].

Nanopolystyrene (100 nm at 1–1000 μg L^−1^) increased the expression of *tdc-1* encoding a tyrosine decarboxylase required for the synthesis of tyramine and decreased the expression of *eat-4* encoding a glutamate transporter. To regulate the nanopolystyrene toxicity in *C. elegans* neurons, the tyramine receptor TYRA-2 acted upstream of MPK-1 signaling. Besides, the toxicity regulation had the contribution of the glutamate receptors, GLR-4 and GLR-8 upstream JNK-1 signaling and DBL-1 signaling, respectively [189].

Epigenetic changes are crucial in mediating the protective response of organisms and *C. elegans* to nanoplastic exposure [190]. Histone acetylation is one of the essential epigenetic regulation mechanisms for controlling gene expression [191]. In *C. elegans*, the exposure to nanopolystyrene (1–100 μg L^−1^) increased the expression of *cbp-1,* which encodes an acetyltransferase, whereas the *cbp-1*(RNAi) worms presented reinforced susceptibility to nanopolystyrene toxicity. During nanopolystyrene exposure, CBP-1 modulated different signaling pathways in the gut (insulin and p38 mitogen-activated protein kinase (MAPK)) and neurons (DAF-7/TGF-β and JNK MAPK). CBP-1 protects from nanopolystyrene toxicity in germline cells by suppressing NHL-2 activity, which modulates insulin communication between the germline and gut [190]. The prolonged exposure to nanopolystyrene (1–100 μg L^−1^) could also affect another critical epigenetic mechanism, the methylation process. Such nanoplastic decreased the expression of *met-2*, an H3K9 methyltransferase. A resistance to nanopolystyrene toxicity could be observed in worms with *met-2* RNAi knockdown in both intestinal and germline cells, followed by an increase in *daf-16*, *bar-1*, and *elt-2* and a decrease in *wrt-3* and *pat-12* gene expressions. That data suggests the role of MET-2-mediated methylation in upstreaming and regulating such genes under nanopolystyrene-induced toxicity [192].

It is well recognized that nanoplastics cause severe multi- and transgenerational toxicity in *C. elegans* [193]. Currently, the researchers have focused on underlying the mechanisms triggering such effects. As aforementioned, toxicity severity is nanoparticle size-dependent [194]. Indeed, the 20 nm nanopolystyrene resulted in more severe transgenerational toxicity than exposure to 100 nm nanopolystyrene at 100 μg L^−1^. The impairments in locomotion and reproduction were detected in the F1–F6 generations for 20 nm nanopolystyrene, whereas the behavioral changes caused by 100 nm nanopolystyrene were only observed in the F1–F3 generations. The authors attributed the oxidative stress activation to increased SOD-3::GFP, HSP-6::GFP, and HSP-4::GFP expressions [195]. The same results in transgenerational toxicity for 30 nm nanopolystyrene (1–100 μg L^−1^) were found in *C. elegans* as brood size and locomotion behavior decreased by generations. Once more, the oxidative stress machinery, including MEV-1, DAF-2, and SOD-3, can induce transgenerational nanopolystyrene toxicity [41]. Moreover, nanopolystyrenes can induce mt UPR in *C. elegans* offspring, which was implicated in *atfs-1*, *dve-1*, and *ubl-5* genes’ regulation [196]. The mt UPR also occurred in parent observations [197].

*C. elegans* miRNA are attributed as regulators facing different kinds of nanomaterial toxicity. In transgenerational studies, the germline *mir-38* expression was reduced after nanopolystyrene exposure (1–100 μg L^−1^), whereas it protected *C. elegans* from toxic effects when overexpressed. The *mir-38* regulation was attributed to the inhibiting activity of downstream targets, including NDK-1, NHL-2, and WRT-3, and at least inhibiting the signaling cascade of NDK-1-KSR-1/2 in worms [198]. *mir-76* also contributed to protection against nanopolystyrene (1–100 μg L^−1^) toxicity in *C. elegans*. GLB-10 functions as a target of *mir-76* in the neurons to regulate nanopolystyrene toxicity. In turn, a signaling cascade of HRG-7-HRG-5 is required to control heme homeostasis, and it is carried out downstream by the neuronal GLB-10. In this way, HRG-5 is responsible for maintaining nanopolystyrene toxicity by inhibiting functions of hypoxia-inducible transcriptional factor HIF-1 and transcriptional factor ELT-2. Those results highlight the *mir-76* role in heme homeostasis-related signaling that regulates the nanopolystyrene toxicity in *C. elegans* [197].

The effects of nanoplastics on ecology, toxicology, and human health are widely discussed in science and legislation, but there are few studies on toxicology using directly sampled nanoplastics from the environment. Most studies use industry-made nanoplastics. There is an opportunity to increase toxicology testing using models such as *C. elegans* to better understand the risks of nanoplastic contamination to the environment and human health.

## 9. Perspectives and Conclusions

Here, we discussed some challenges and the main advantages of using the *C. elegans* model for nano-based strategies. The biomedical efficacy of nanotechnology-based strategies is still dependent upon post-formulation features (e.g., stability, solubility, clearance, cytotoxicity, and genotoxicity), which implies industrial applications that follow regulatory processes.

*C. elegans* has been extensively explored in nanotechnology due to its rapid development and lifespan, which confer speed in assay execution. Indeed, it is one of the most used models in multi- and transgenerational studies. In different body parts, the labeled nanomaterial can be easily viewed because of the nematode’s transparency. This should be considered a first step in the synthesis process since the nanoparticle properties can change after the dye has been incorporated into the molecule. In agriculture, nanopesticides provide slow, controlled, and sustained active ingredient release, increasing product shelf life and improving water solubility. Furthermore, nanotechnology in this field reduces the environmental impact of pesticides. However, the high costs and difficulties of transposing to an industrial scale are still limiting factors for the wide use of nanopesticides.

The possibility of improving drugs, plant extracts, oils, and even pesticides can be tested in *C. elegans*. Organic nanoparticles provide the additional advantage of being orally administered in *C. elegans*, beyond the improvements in the bioavailability of compounds as carrier tools. The low toxicity of organic nanoparticles applied to *C. elegans* with biomedical aims makes them a suitable alternative to nanotechnology used in experiments. The carbon nanotubes can be absorbed by cells and used to deliver vaccines, drugs, or protein carriers. *C. elegans* showed behavioral, transcriptomic, proteomic, and physiological changes after exposure to carbon nanotubes, and this alerts us to the importance of further studies to elucidate such toxicological effects and the impacts on the future medical use of carbon nanotubes.

Using environmental samples of nanomaterials or even macro/microplastic samples in the environment that are transformed into nanoplastic in the laboratory is a promising future in nanoplastics and *C. elegans* studies. The ability of nanoplastics to release additives and/or adsorb other hydrophobic organic contaminants, such as pesticides, is crucial for using environmental samples to perform toxicological studies.

*C. elegans* can respond to most nanomaterials and can be a feasible biosensor for early monitoring of such compounds. The outcomes can boost the investigations in more complex animals and benefit human health.

## Figures and Tables

**Table 1:** Composition and size features of some nanoparticles used in nanomedicine. Adapted from [15].

Nanoparticles	Size Range (nm)	Applications
Silver	2–20	Antibacterial activity
Gold (spheres and rods)	2–150	Site-specific imaging and hyperthermia
Titanium dioxide	5–50	Ultraviolet radiation protection (sunscreen transparent on skin)
Iron oxide	10–250	MRI, site-specific imaging, and magnetic hyperthermia
Silica (mesoposous)	20–300	Drug delivery and tumor targeting
Carbon (fullerenes and nanotubes)	1–40	MRI contrast and antioxidants
Quantum dots (core/shell)	2–20	Fluorescence imaging
Copolymers agents	<200	Drug and gene delivery
Dendrimer-based conjugates	2–50	Drug delivery and targeting
Exosomes (vesicles)	30–90	Regenerative therapy
Liposomes	50–350	Drug and gene delivery

MRI—magnetic resonance imaging.

**Table 2:** Upregulated and downregulated genes of nematodes exposed to PAMAM.

Metabolic Pathways	No. of Active Genes	PAMAM
Cytoplasmic ribossomal proteins	63	*rpl-6* (3.11) *rpl-10 * (3.11) *rpl-13* (−4.93) *rpl-15* (9.31) *rpl-17* (7.31) *rpl-18* (−12.67) *rpl-26* (2.87) *rpl-32* (−2.74) *rpl-43* (−7.90) *rps-4 * (2.25) *rps-5* (5.42) *rps-9* (−3.38) *rps-12* (2.12) *rps-13* (−5.57) *rps-14* (1.04) *rps-17* (2.06) *rps-30* (−2.98)
Glycolysis	25	*hxk-1* (3.07) *acl-4* (−6.67) *fbp-1* (−3.67) *aldo-2* (4.10) *pyc-1* (3.07) *pck-1* (2.27)
DNA replication	25	*orc-1* (−4.99) *mcm-3* (3.17) *mcm-2* (−18.54) *arpa* (2.06) *rfc-1* (−1.04) *f10c2.4 * (2.69) *cdc-6* (2.04)
LIN-12-Notch lateral signaling	15	*let-23* (2.04) *lst-2* (2.89) *dpy-23* (2.19) *mpk-1* (2.79) *unc-101* (2.02)
Sex determination	17	*sex-1* (−2.89) *sea-1* (7.70) *sdc-3* (1.04) *fem-1* (3.00) *sel-10* (2.03)
Translation factors	31	*eif-3.H* (−2.08) *eif-3.G* (−5.72) *ife-3* (2.67) *eef-1g* (−2.13) *eft-2* (−7.23) *eif-2α* (−2.03) *eif-3b* (2.20)
**Total number of genes** **(+ upregulated, − downregulated)**		47 (+29, −18)

Blue: upregulated. Red: downregulated. Source: adapted from [112].

## Data Availability

Not applicable.

## Abbreviations

3-MA	3-methyladenine
*C. elegans*	*Caenorhabditis elegans*
CAT	Catalase
COVID-19	Coronavirus disease
*E. coli*	*Escherichia coli*
EMA	European Medicines Agency
FDA	Food and Drugs Agency
fMWNTs	Acid-functionalized MWCNTs
GRK2	G protein-coupled receptor kinase
HDFM	Hyperspectral dark field microscopy
HIV/AIDS	Human immunodeficiency virus/acquired immunodeficiency syndrome
MAPK	Mitogen-activated protein kinase
MNPs	Magnetic nanoparticles
MRI	Magnetic resonance imaging
mRNA	Messenger ribonucleic acid
mt UPR	Mitochondrial unfolded protein response
MWCNTs	Multiwalled carbon nanotubes
N-GQD	Nitrogen-doped graphene quantum dots
NAC	N-Acetyl-L-cysteine
NIR	Near-infrared
PAMAM	Poly(amidoamine) dendrimers
PEG	Polyethylene glycol
PLA	Polymeric polylactic acid
PRDx	Peroxiredoxin-2
ROS	Reactive oxygen species
SAR	Specific absorption rate
SOD	Superoxide dismutase
SWCNTs	Single-walled carbon nanotubes
TEM	Transmission electron microscopy
μ-SRXRF	Synchrotron radiation X-ray fluorescence
